# Early-Warning Measures for Ecological Security in the Qinghai Alpine Agricultural Area

**DOI:** 10.3390/ijerph17249292

**Published:** 2020-12-11

**Authors:** Jing Guo, Zhen Wei, Jun Ren, Zenghai Luo, Huakun Zhou

**Affiliations:** 1 Key Laboratory of Restoration Ecology for Cold Regions in Qinghai, Northwest Institute of Plateau Biology, Chinese Academy of Science, Xining 810008, China; guojing@nwipb.cas.cn; 2 Research Department of Ecological Environment, Qinghai Academy of Social Sciences, Xining 810000, China; 3 University of Chinese Academy of Sciences, Beijing 100049, China; 4 Research Department of Economics, Qinghai Academy of Social Sciences, Xining 810000, China; skyweizhen@126.com; 5 Graduate School of Qinghai University, Qinghai University, Xining 810016, China; Renjun@cugb.edu.cn; 6 Qinghai General Station of Animal Husbandry, Xining 810003, China; luozenghai@163.com

**Keywords:** entropy method, Qinghai, alpine agricultural mountain area, ecological security warning

## Abstract

The study area of this paper is the Qinghai alpine agricultural mountain area. An ecological security early-warning model is used to identify the early warning signs of ecosystem destruction, environmental pollution and resource depletion in districts and counties from 2011 to 2018. A combination of qualitative and quantitative early-warning models is used to predict the existence of hidden or sudden advance warnings. The grey (1, 1) model (GM) is used to predict the evolution trend of ecological security warning situations from 2019 to 2021. On this basis, GIS technology is used to analyze the spatial pattern changes in three periods. The results show that from 2011 to 2018, the ecological environment in Qinghai’s alpine agricultural mountainous area gradually improved. In 2018, the ecological security early-warning values of all districts and counties were greater than the 2011 values. However, in 2018, the ecological security early-warning levels of PA, LD and HZh (PA, LD and HZh refer to Ledu District, Ping’an District and Huzhu Tu Autonomous County respectively.) were in the “good” ecological early-warning state, while the ecological security levels of other cities were still in the “moderate” or “mild” ecological warning state. According to the prediction results, the early-warning level of ecological security in Qinghai’s alpine agricultural mountainous areas will improve further in 2021, with the “good” states dominating. From a spatial perspective, the ecological environment in the northeast region is better than that in the southern region, and the internal differences in the ecological security early-warning levels tend to narrow. Thus, we propose that areas with different ecological security levels should focus on the management and protection of the ecological environment or carry out ecological restoration or reconstruction. The aim of this paper is to provide a reference for the improvement of the ecological environment in general and the sustainable development of the economy and society as well as the ecological environment of alpine agricultural mountainous areas in particular.

## 1. Introduction

Since the beginning of the 21st century, with the rapid development of industrialization, urbanization, and the population, the contradiction between population development and ecological stability of land has become increasingly acute. There has been a generally deteriorating trend in ecology and the environment, and the problem of ecological security has become a new challenge to the sustainable development of human society [1]. An important aspect of the ecological security research [2,3] involves the study of ecological environmental early-warnings, which seeks to identify warning signs of ecosystem degradation, environmental quality deterioration and resource depletion caused by human activities. A combination of qualitative and quantitative early-warning modes is used to predict imminent emergency situations in advance and to propose timely warnings to achieve the goal of protecting the ecological environment [4,5].

The content of the ecological security early-warning research includes two major categories: theoretical and empirical research and ecological risk assessment and prediction. The theoretical research mainly focuses on the analysis of ecological early-warning concepts and the selection of indicators. For example, Fu Bojie identified evaluation indicators based on natural resources, social development status, and economic development level to carry out early-warning research on the quality of China’s ecological environment [6]. Another example is Chen Guojie, who proposed mathematical models of environmental early-warning such as the critical point warning, an approach that addresses environments with slower degradation rates [4]. At the empirical level, different research methods and technologies are combined, for example, the ecological security prediction model established by the grey prediction model (the dynamic predictive model of grey system theory, that is, the mathematical model of simulation synthesis obtained from data characterizing system behavior) [7], and the Markov prediction method, which is used to predict ecological security from the perspective policing [8]. The time and space prediction analysis of desertification disasters [9], based on GIS technology monitoring, can be combined with GIS technology to analyze changes in the spatial patterns of such disasters [10]. Ecological risk assessment and prediction mainly include the monitoring of early-warning sources, early-warning environment and early-warning signals [11]. For example, Tegler and others further applied core monitoring variables to early-warning systems [12]. Parr et al. supplemented the monitoring of early-warning sources in early-warning monitoring systems and regarded human factors as an important parameter of the early-warning environment [13]. Dakos used system theory to analyze STPA (System-Theoretic Process Analysis) to identify early-warning signals [14]. Bolton simulated the early warning signs of the demise of the Amazon rainforest [15], and Dan implemented a new environmental monitoring and early-warning system for the western Black Sea [16]. Mitra et al. conducted an ecosystem stability assessment by using a damped drive pendulum, the Amazon rainforest model (as a known climate tilt element) and the Daisyworld model [17].

The grey forecast model is a forecasting method that uses a small amount of incomplete information to establish a mathematical model and make predictions. This type of prediction is based on past and present development laws of objective things with the aid of scientific methods to describe and analyze future development trends and conditions and to form scientific assumptions and judgments. The research fields that employ the grey prediction model mainly involve different ecosystems, watersheds [18], wetlands and ecologically fragile areas [19], wetlands [20], nature reserves [21], arid areas [22], and cities [23]. Ecological security early-warning methods mainly include the following: the grey (1, 1) model (GM) [24], the GM (1, 1) unbiased model [25], the BP(Back Propagation) neural network model [26], classification trees [27], the RBF Neural network model [28], the Markov model [29], the scenario analysis model [30], the regulation analysis model [31], the system dynamics model [32] and cellular automata. [33]. However, compared with retrospective evaluation, the research on environmental impact assessment is still relatively undeveloped, mainly due to the poor adaptability of prediction models, single early-warning methods, and varied accuracy of early-warning results. In addition, there is as yet no accurate prediction error correction method [3], which not only restricts the development of the early-warning research but also provides an entry point for future early-warning research.

This paper synthesizes existing studies to build a framework based on population, ecological environment, socioeconomic resources and natural resources (PESN) to apply the grey early-warning model to the county-level modeling method to determine the complex interaction, impact and feedback between the alpine agricultural mountainous area and the related land-use change and ecological environment. The model is based on the existing methods employed to realize early-warnings with regard to ecological security in agricultural mountainous areas in 2020. The modeling method was applied to the alpine agricultural mountainous area of Qinghai Province. We chose this area as a case study because the population of the region is expected to grow by approximately 15% from 2011 to 2020, and the socioeconomic development of the region is rapid; thus, the demand for natural resources in the region is very high. This condition will likely have a negative impact on the ecological environment. Qinghai Province is located in the northeast of the Qinghai Tibet Plateau. It has unique resource advantages. Lancang River is the birthplace of the Yangtze River, and its ecological environment is relatively fragile. There are some problems in the Qinghai alpine agricultural mountainous areas such as a simple ecosystem structure, poor self-regulation ability, with a low impact on natural disasters and human activities. The case study involves the following main research questions: What will be the status of ecological security and development trends in the next few years? Where will ecological security warnings mainly be distributed in the areas of the Qinghai alpine agricultural mountainous area? These identified sites are where ecological security management and supervision should be strengthened in the future.

Generally speaking, the research on ecological safety early-warning system is still developing, there are gaps, and the current evaluation process is relatively simple. In particular, the ecological safety early-warning system and the GM (1, 1) are combined and used in the same case study; and most of the existing studies are concentrated on the overall regional scale, rarely involving internal districts and counties [34]. Based on the existing literature, this paper firstly constructs the Qinghai Alpine Agricultural and Mountainous Area Ecological Security Early-warning Index System (PESN), including population, ecological environment, socio-economic and natural resources subsystems; secondly, we propose the early-warning interval and level of this indicator. The division value is studied; finally, based on the GM (1, 1) and GIS technology, we analyze the recent spatial pattern changes of ecological security early-warning. In addition, the hybrid model overcomes the defects of using only a single indicator and low accuracy of traditional models, and the purpose of using the GM (1, 1) as a predictive model is its simplicity, high accuracy, and no need for typical probability distributions [35]. Compared with similar studies [36,37], this article is based on the double test of residual error and posterior error to improve the accuracy of ecological security early-warning value and model accuracy. The study emphasizes the importance of alpine agricultural mountainous areas to the development of ecological security, and provides references for comprehensive management of the local ecological environment and improving the sustainability of land use and ecological protection policies.

This model can also promote further research development and knowledge advancement regarding the socioeconomic interaction of land-use change and ecological environment change [38]. The method applied in this study can be used in the quantitative study of the ecological security system of other agricultural mountainous areas, and its basis provides feedback and more realistic accounting of the impact on land use.

## 2. Study Area, Data Sources and Evaluation Index System

### 2.1. Study Area

**Figure 1:**
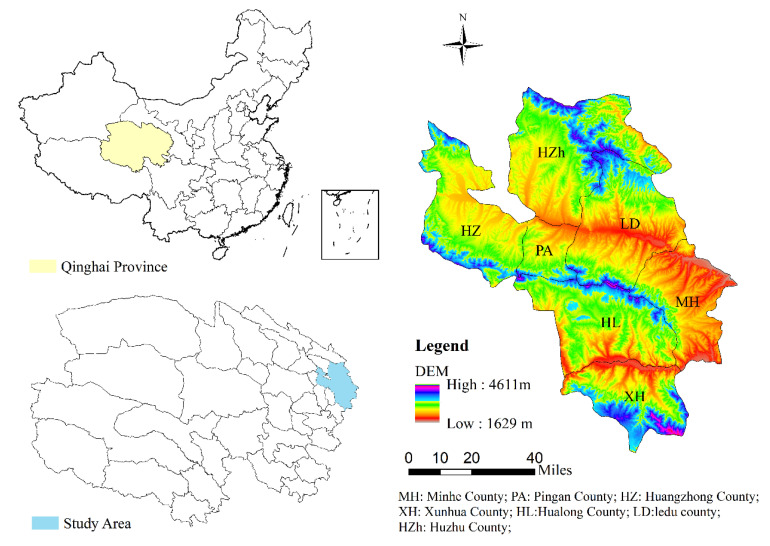
Location of a study on ecological security in the Qinghai–Tibet Plateau of the Qinghai alpine agricultural area.

This study selects the Qinghai Hehuang Valley, which is an important agricultural production area in the alpine region, as its representative case. Specific administrative areas include Huangzhong County (HZ), Ledu District (LD), the Ping’an District (PA), Huzhu Tu Autonomous County (HZh), Minhe Hui Autonomous County (MH), Hualong Hui Autonomous County (HL), and Xunhua Salar Autonomous County (XH), representing 2 districts and 5 counties (Figure 1). The study area covers approximately 15,900 km^2^ and a population of approximately 1.9172 million.

The study area is located in the ecotone of the Qinghai–Tibet Plateau and the Loess Plateau. The mountains and rivers are intertwined, the gullies are vertical and horizontal, and the terrain is complex. The altitude is between 1650 and 4635.5 m [39], and the region is a typical alpine agricultural mountainous area. The area has sufficient sunlight and strong solar radiation. The annual precipitation is 164.3–531.9 mm, and the annual average temperature is 3.8–18.6 °C. There are large regional differences and a semiarid continental climate. Due to natural, geographic, societal, and historical limitations, most of the Qinghai Province consists of underdeveloped areas, with low development levels and low social and economic development levels. The total arable land area in the province is approximately 58.94 × 10^4^ ha and is mainly concentrated in the Hehuang Valley, which is an arid valley in the traverse mountain area and oasis on the edge of Qaidam land. The ecological security of the alpine agricultural mountainous area plays an important role in promoting the development strategy of “establishing an ecological province” in Qinghai Province. At the same time, most of the alpine agricultural mountainous areas are located in extremely poor areas with large populations that face severe population and resource pressures for which economic and environmental coordination is extremely difficult. The site is typical and appropriate for the study of alpine ecological vulnerability. The objectives of this study are to protect the ecological security of the Qinghai–Tibet Plateau, to fulfill the responsibility of maintaining ecological security and to achieve the goal of sustainable development to provide a scientific basis for the economic development and ecological security construction of alpine agricultural mountainous areas in this new stage of development.

### 2.2. Data Sources

The index data involved in this study came from the Statistics Bureau, Environmental Protection Bureau and Poverty Alleviation Bureau of each city, district and county. Specifically, the data were derived from the “Haidong Statistical Yearbook (2011–2018)” [40,41,42,43,44,45,46,47], “Xining Statistical Yearbook (2011–2018)” [48,49,50,51,52,53,54,55], “Huangzhong Statistical Yearbook (2011–2018)” [56,57,58,59,60,61,62,63], “Ledu Statistical Yearbook (2011–2018)” [64,65,66,67,68,69,70,71], “Qinghai Province National Economic Statistics of Mutual Assistance Tu Autonomous County (2011–2018)” [72,73,74,75,76,77,78,79], “National Economic and Social Development Statistics of Minhe Tu Autonomous County (2011–2018)” [80,81,82,83,84,85,86,87] and other county statistical yearbooks. Among them, population, socioeconomic, and natural resource data mainly come from statistical yearbooks. Except for fertilizer application intensity and pesticide application intensity from statistical yearbooks, other ecological environment data come from statistical bureaus. The missing data for individual years were supplemented by interpolation of adjacent years. In addition, some data came from secondary sources such as articles and reports. The spatial data came from the geospatial data cloud (http://www.gscloud.CN/), and, after scanning, the registration was vectorized in ArcGIS 10.2 software (Environmental Systems Research Institue, Redlands, CA, USA).

### 2.3. The Evaluation Index System

An early-warning index system is based on a comprehensive and complex dynamic process. In recent years, researchers have tended to build scientific and reasonable index systems to develop early-warning systems for regional environments, biology and ecosystem security with specific index selection. Therefore, in this context, on the basis of expanding the existing research results, this paper proposes a PESN ecological security early-warning system for agricultural mountainous areas that comprehensively evaluates the ecological security development level of the Qinghai Tibet Alpine agricultural mountainous areas based on the four dimensions of population, ecological environment, social economy and natural resources. Population early-warning mainly refers to the early-warning of population quantity and population quality. Due to the strong continuity of population development, if one generation does not develop well, it will often affect the quality of life and environmental conditions of later generations. An ecological environment early-warning mainly refers to the pollution impact of human activities on the environment and timely warnings in terms of environmental quality impacts on the reverse succession of the ecosystem. Social and economic early-warnings mainly refer to warnings regarding the sustainable development of the social economy. Natural resources early-warnings are mainly used to forewarn regarding changes in the resource background value of life support system elements.

## 3. Study Methods

### 3.1. Ecological Security Early-Warning Model

- Step 1: Ecological Safety Early-warning Indicator Selection

(1) Index selection principle

The ecological safety early-warning system mainly solves five basic problems [88]: (i) Ecological status monitoring, which mainly monitors the changes in ecosystems at different time and space scales, changes in internal ecosystem factors, the degree of ecological risk in the monitored area, and changes in man-made landscapes and the effects of different natural and human factors; (ii) The monitoring of ecosystem impacts, pollution caused by different types of economic activities, and consumption of biological resources; (iii) The monitoring of extraordinary situations, including the monitoring of technical accidents and natural disasters of different objects, the monitoring of increased solar activity, floods, storms, typhoons, earthquakes, and mudslides; (iv) Resource safety monitoring for the development and utilization of natural resources; (v) The monitoring of basic earth sciences such as meteorology, climate, and oceans.

Therefore, regional ecological security early-warnings aim at regional sustainable development, predict changes in the ecological environment from time and space scales, and identify relevant aspects of population, nature, the ecological environment, and the social economy in the evaluation of ecological security quality. Indicator factors include bearing capacity, stability, productivity, buffer capacity and control capacity to analyze the ability of a region’s sustainable development [6].

(2) Index Selection

According to the basic principles of regional ecological security early-warning and the principle of index selection, the regional carrying capacity, stability, productivity, buffer capacity and control capacity are implemented into specific indicators. After theoretical analysis and empirical judgment, starting with the structural analysis of regional population, nature, ecological environment and socio-economic subsystem, the following indicators are selected as the early-warning indicators of regional ecological security. When conducting early-warning research on ecological security in a certain area, these factors can be increased or decreased in combination with the characteristics of the area.

(i) Population indicators: population density, natural population growth rate, population carrying rate, nine-year compulsory education consolidation rate, proportion of rural employees, quality of agricultural workers.
(ii) Eco-environmental indicators: the treatment rate of industrial wastes, the proportion of the protected area in the land area, the rate of natural disasters, the restoration rate of degraded land, the biological abundance index, the human interference index, the area and intensity of soil erosion.
(iii) Socioeconomic indicators: GDP per capita, annual growth rate of GDP, annual net income per capita, proportion of tertiary industry in GDP, economic density, proportion of environmental protection investment in GDP, economic output per unit of land.
(iv) Natural resource indicators: per capita arable land area, forest coverage, per capita water resources, per capita forest area, effective irrigation area.

- Step 2: Dimension Reduction

First, the dimensionless treatment should be carried out on the characteristic values of the indicators. Based on the characteristics of the ecological security early-warning factors, there are two types of indicators: developmental (positive) indicators and restrictive (negative) indicators. $X_{\mathrm{ij}}$ is the jth indicator for the ith area. Converted $X_{\mathrm{ij}}$ into relative data with values in the range [0, 1]. It is calculated as follows:

Developmental (positive) indicators:

(1) $$y_{\mathrm{ij}}=\frac{X_{\mathrm{ij}}-X_{\mathrm{jmin}}}{X_{\mathrm{jmax}}-X_{\mathrm{jmin}}},\;\left(i=1,2\cdots m;\;j=1,2,n\right)$$

Restrictive (negative) indicators:

(2) $${\;y}_{\mathrm{ij}}=\frac{X_{\mathrm{jmax}}-X_{\mathrm{ij}}}{X_{\mathrm{jmax}}-X_{\mathrm{jmin}}},\left(i=1,2\cdots m;\;j=1,2\cdots n\right)$$

where $X_{\min}$ is the minimum performance measure for all alternatives against the jth indicator. $X_{\max}$ is the maximum performance measure for all alternatives against the jth indicator. “Positive index” (Formula (1)) refers to the index where more is better, indicating a higher level of ecological security. The negative index (Formula (2)) refers to the index where a higher number is worse, indicating a higher level of ecological security.

- Step 3: Weight and Early-warning Value

To ensure that the research results are objective and accurate and to attempt to eliminate human interference in the calculation of the indicator weights, this study used the entropy method to determine the weights of the early-warning indicators. In information theory, entropy reflects the degree of information disorder and can be used to measure the amount of information [89]. The more information an indicator carries, the greater its effect on decision-making [90]. According to step 2, all the original data are dimensionless and the weight of each evaluation index is obtained through the entropy weight method. It is calculated as follows.

According to the definition of entropy, where m is the number of evaluation objects and n is the number of evaluation indicators, the entropy of evaluation indexes can be determined as follows:

(3) $${ℯ}_{j}=\frac{1}{\ln n}\sum_{i=1}^{m}f_{\mathrm{ij}}\ln f_{\mathrm{ij}}$$

(4) $$f_{\mathrm{ij}}=y_{\mathrm{ij}}/\sum_{j=1}^{n}y_{\mathrm{ij}}$$

where ${ℯ}_{j}$ is the entropy of the ith index and $f_{\mathrm{ij}}$ is the standardized index data. If $f_{\mathrm{ij}}$ = 0, then use 0.0001 instead of the calculation, and the entropy weight of the ith index can be defined as:

(5) $$W_{i}=b_{i}/\sum_{i}^{m}b_{i}$$

where $W_{i}$ is the weight of index i; $b_{i}$ is the difference coefficient of index $X_{i}$, and $b_{i}$ = 1−${ℯ}_{j}$.

- Step 4: Ecological Security Early-warning Value

The product of the weight of the jth index and the standard value ${X_{\mathrm{ij}}}^{\prime }$ of the  jth evaluation index in the ith area in the standardization matrix is taken as the evaluation value $P_{\mathrm{ij}}$ of $X_{\mathrm{ij}}$:

(6) $$P_{\mathrm{ij}}=W_{j}{X_{\mathrm{ij}}}^{\prime }$$

Finally, the warning value for the ith area is as follows:

(7) $$P_{i}=\sum_{j=1}^{n}P_{\mathrm{ij}}$$

Obviously, the larger the $P_{i}$, the better the effect of the sample. Finally, the evaluation conclusion can be obtained by comparing all the values of $P_{i}$.

- Step 5: Ecological Security Early-Warning Criterion

The selection of ecological security early-warning standards must reflect the scope and degree of ecological security. This study refers to national, industrial and international standards, regional background research values, and analogy standards. Based on the reference literature [10,91,92,93], the evaluation criteria of ecological security early-warning in 7 areas of the Qinghai alpine agricultural mountainous area were divided into five grades: “Huge alarm”, “Moderate warning”, “Light warning”, “Good” and “Excellent” (Table 1). Different early-warning values correspond to different levels of safety based on early-warning conditions and are scored between 0 and 1. The closer the score is to 1, the more ideal the ecological security status is; the closer the score is to 0, the worse the regional ecological security status is, and the greater the hidden dangers [94].

### 3.2. The Grey (1, 1) Model

Grey system theory was founded in the 1980s and was developed by Deng Julong, a famous mathematician in China [95]. Grey system theory essentially established a new structural system. Its main content includes a theoretical system based on a grey algebra system, grey equation, and grey matrix. Its method system is based on the generation of grey sequences and an analysis system based on grey relational spaces. The grey model (GM) is the core model system, and its main technical systems include system analysis, evaluation, modeling, prediction, decision-making, control, and optimization. GM (1, 1) is the most commonly used grey forecasting model. The basic function of the GM (1, 1) model is to fully develop and utilize the explicit and implicit information in the existing data, and the randomness existing in a given series is cumulatively weakened. Revealing the regularity of data allows the new sequence to reflect the trend of the original sequence, which can be used to study the future temporal distribution of specific time intervals [96,97]. Grey system theory is an important method for studying discrete data series with small numbers of samples and incomplete information [98]. Therefore, it is widely used in the prediction of ecological safety indexes. The main modeling steps are as follows.

The GM (1, 1) model only uses the system behavior data sequence to establish predictions, which is a relatively simple and practical single sequence modeling method. In the case of time series data, only regular time variables are involved; in the case of horizontal series data, only regular object number variables are involved, and no other explanatory variables are involved. The essence of the model is a modeling method that is relatively simple to apply and can reveal information about development and change of practical value.

- Step 6: GM (1, 1) Model Construction

(i) Sequence generation by accumulation

The accumulation generation number 1-AGO (the first-order accumulating generation) stands for a single operation. The original sequence is $X^{\left(0\right)}=\{x^{\left(0\right)}(1),x^{\left(0\right)}(2),\ldots ,x^{\left(0\right)}(n)\}$, and the sequence after the single operation is as follows:

(8) $$X^{\left(1\right)}=\{x^{\left(1\right)}(1),x^{\left(1\right)}(2),\ldots ,x^{\left(1\right)}(n)\}$$

where

(9) $$x^{\left(1\right)}(k)=\sum_{i=0}^{k}x^{\left(0\right)}(i)=x^{\left(1\right)}(k-1)+x^{\left(0\right)}(k)$$

Then, the mean series is calculated as follows:

(10) $$z^{\left(1\right)}(k)=0.5x^{\left(1\right)}\left(k\right)+0.5x^{\left(1\right)}\left(k-1\right),k=2,3,\ldots ,n.$$

(ii) Construction of the accumulation matrix *B* and the constant vector $Y_{n}$

(11) $$B=\left[\begin{array}{cc} -z^{\left(1\right)}(2) & 1\\ -z^{\left(1\right)}(3) & 1\\ \ldots & \ldots \\ -z^{\left(1\right)}(n) & 1 \end{array}\right]Y_{n}=\left[\begin{array}{c} x^{\left(0\right)}(2)\\ x^{\left(0\right)}(3)\\ \ldots \\ x^{\left(0\right)}(n) \end{array}\right]$$

(iii) Determination of the least-squares solution to the grey parameter vector $\overset{\wedge }{\alpha }$

Using this series, the first-order differential equation based on a single variable is established and used as the prediction model (that is, the GM (1, 1) model). The standard form of the grey difference equation is as follows:

(12) $$x^{\left(0\right)}(k)+az^{\left(1\right)}(k)=b,k=2,3,\ldots ,n.$$

The corresponding whitening differential equation is as follows:

(13) $$\frac{dx^{\left(1\right)}(t)}{dt}+ax^{\left(1\right)}(t)=b$$

where *a* and *b* are the development coefficient of the system and the endogenous control of grey scale, respectively.

The estimation formula for the parameter vector ab can be written in the following form:

(14) $$\hat{\Phi }={\left[\hat{a},\hat{b}\right]}^{T}={\left(B^{T}B\right)}^{-1}B^{T}Y_{n}$$

(iv) Substitution of the parameter into the sequence after the single accumulation

The time–response function of the GM (1, 1) model is as follows:

(15) $${\hat{x}}^{\left(1\right)}(k+1)=\left[x^{\left(0\right)}\left(1\right)-\frac{b}{a}\right]e^{-ak}+\frac{b}{a}\;k=1,2,\ldots ,n$$

(v) Retrieval of the reduction value

The recovered data $x^{\left(0\right)}(k+1)$ can be retrieved by the inverse accumulated generating operation:

(16) $${\hat{x}}^{\left(0\right)}(k+1)={\hat{x}}^{\left(1\right)}(k+1)-{\hat{x}}^{\left(1\right)}(k);{\hat{x}}^{\left(0\right)}(1)=x^{\left(1\right)}(1)$$

(17) $$\mathrm{or}\;{\hat{x}}^{\left(0\right)}(k+1)=-a\left(X^{\left(0\right)}\left(1\right)-\frac{b}{a}\right)e^{-ak}$$

(vi) Calculation of the residual error and the relative error

(18) $$\varepsilon^{\left(0\right)}(k)=X^{\left(0\right)}\left(k\right)-{\hat{X}}^{\left(0\right)}\left(k\right)$$

(19) $$e(k)=\varepsilon^{\left(0\right)}\left(k\right)/X^{\left(0\right)}\left(k\right)$$

where $\varepsilon^{\left(0\right)}(k)$ is the residual error and e(k) is the relative error.

- Step 7: GM (1, 1) Model Accuracy Test

(vii) Posterior error test

A common method used to evaluate the grey model is the posterior error test. This method tests the statistical characteristics of the residual error distribution, and the posterior error ratio C and the small error p are used to evaluate the model.

Posterior error ratio: $C=\frac{S_{2}}{S_{1}}$, small error probability: $p=p\left\{\left|e\left(k-\bar{e}\right)\right|\right\}<0.6745S_{1}$, where

(20) $$S_{1}^{2}=\frac{1}{n}{\sum_{k=1}^{n}\left(x^{\left(0\right)}\left(k\right)-x^{-\left(0\right)}\right)}^{2}$$

(21) $$S_{2}^{2}=\frac{1}{n}{\sum_{k=1}^{n}\left(e\left(k\right)-\bar{e}\right)}^{2}$$

(22) $$x^{-(0)}=\frac{1}{n}\sum_{k=1}^{n}\left(x^{\left(0\right)}\left(k\right)\right)$$

(23) $$\bar{e}=\frac{1}{n}\sum_{k=1}^{n}e\left(k\right)$$

The levels of model accuracy are presented in the following table (Table 2).

(viii) Residual test

Calculate ${\hat{X}}^{\left(1\right)}(i)$ according to the prediction model, and accumulate and subtract ${\hat{X}}^{\left(1\right)}(i)$ to generate ${\hat{X}}^{\left(0\right)}(i)$, and then calculate the absolute error series and relative error series of the original series $X^{\left(0\right)}(i)$ and ${\hat{X}}^{\left(0\right)}(i)$.

(24) $$\Delta^{\left(0\right)}(i)=\left|X^{\left(0\right)}\left(i\right)-{\hat{X}}^{\left(0\right)}\left(i\right)\right|\left(i=1,2,\cdots ,n\right)$$

(25) $$\Phi (i)=\frac{\Delta^{\left(0\right)}\left(i\right)}{X^{\left(0\right)}\left(i\right)}\times 100\%\left(i=1,2,\cdots ,n\right)$$

If the residual test and posterior error test can pass, it means that the model accuracy calibration can be used for prediction.

**Figure 2:**
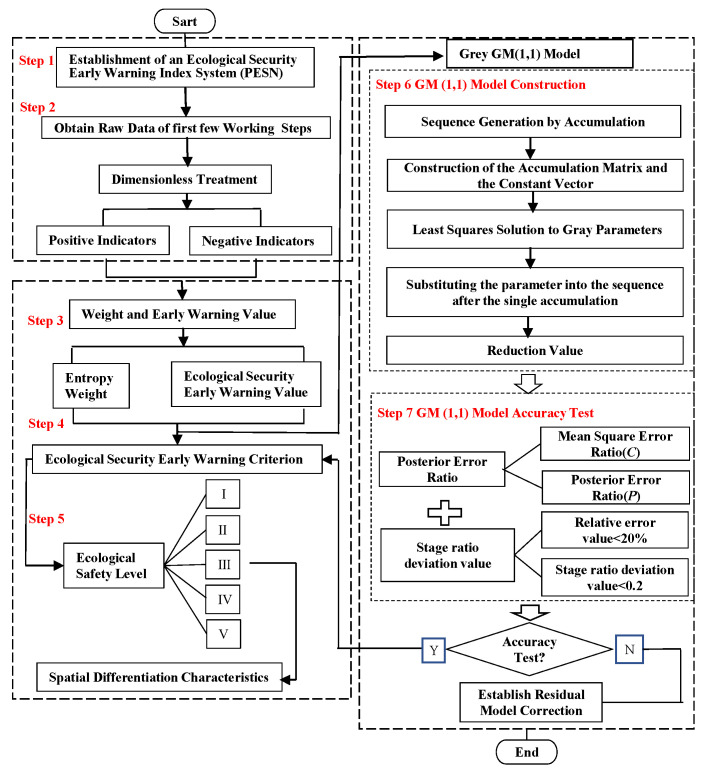
Model flow framework.

In order to make the modeling steps in the research method easier for readers to understand, a framework diagram of the model process is drawn, as shown in Figure 2.

### 3.3. The Accuracy of the Data Realization Model

In order to ensure the accuracy of the early-warning results, the accuracy of the model must be tested. In this study, the combination of the posterior error test and residual error test has improved the accuracy of the model to a certain extent. Based on the data from 2011 to 2018, the ecological safety early-warning value of each district and county in 8 years was calculated according to the operation steps 2–6 in the research method. According to the inspection method in step 7, the model accuracy inspection results are shown in Table 3. From the table, the C value and *p* value of the posterior difference test both reached the middle or above grade; the average relative error was 1.61% to 5.80%, and the absolute value of the deviation value of the grade ratio was less than 0.2. It shows that the models in all districts and counties have reached the qualified standard, and the accuracy is accurate for the next prediction. Based on the results and the prediction formula ${\hat{x}}^{\left(0\right)}(k+1)=-a\left(X^{\left(0\right)}\left(1\right)-\frac{b}{a}\right)e^{-ak}$, the early-warning values for each district and county from 2019 to 2021 are predicted, and the prediction results for 2021 are shown in spatial differentiation. The early-warning model has certain effects and can be used for emergency response.

## 4. Results and Analysis

### 4.1. Establishment of an Ecological Security Early-Warning Index System

The ecological security early-warning system of the Qinghai alpine agricultural mountainous area forecasts the current status and development trend of ecological safety in the Qinghai alpine agricultural mountainous area. According to the safety threshold, the disturbance of the ecosystem is measured, and the corresponding discrimination is made to carry out ecological safety early-warning for various systems of the local population, ecological environment, social economy and natural resources. The quantitative calculation of the alarm conditions of each system through the model can provide comprehensive ecological security early-warning for the alpine agricultural mountainous environment. The ecological security early-warning system is a composite system covering population-society-economic-nature. Therefore, the final indicator system includes 4 types of systems (population early-warning system, ecological environment early-warning system, natural resource early-warning system and socioeconomic early-warning system) and 20 indexes that can reflect the early-warning indicators of ecological security in alpine agricultural mountainous areas (Table 4). According to the above formula, the type indicator weight and comprehensive indicator weight of 20 indexes were calculated (Table 4).

### 4.2. Analysis of the Comprehensive Trend of Ecological Security Early-Warning Value

Table 5 shows that the ecological security early-warning values of all districts and counties in the Qinghai alpine agricultural mountainous area were higher in 2018 than in 2011, indicating that the overall ecological environment in the mountainous area gradually improved. In 2011, only the ecological security early-warning value of PA was between 0.4 and 0.6, and the level was in the light warning state of level III. This result shows that the ecological environment of these areas is in a poor state, the ecosystem has suffered great damage, the ecosystem structure is incomplete, and the service function is degraded; however, the basic function can be maintained. Compared with 2011, the ecological security of the region improved markedly by 2018. The early-warning value of HZ, PA and LD ecological security was between 0.6008 and 0.6448, and ecological security was at level IV, which is one or two levels higher than that in 2011. The early-warning value of the ecological security of PA, which had the best ecological status among all districts and counties, reached 0.6448, which was at level IV. During the 8-year period, the overall ecological environment in Qinghai’s alpine agricultural mountainous areas gradually improved. In terms of the early-warning values of ecological security, the ecological environments of all districts and counties have improved, and in terms of ecological security levels, the ecological security levels of most regions have improved.

From the perspective of changes in the ecological security early-warning rankings of various districts and counties, the ecological security early-warning rankings of various districts and counties have changed remarkably in the past 8 years. PA, LD and HZh rank in the top three. The rankings of the remaining districts and counties have fluctuated greatly over time. Among them, XH dropped from second place in 2011 to seventh in 2017, and the ranking of XH showed a downward trend year by year. HZ rose from sixth place in 2011 to fourth place in 2012, and it has remained in fourth place ever since. MH’s ranking has experienced a “bottom-up” fluctuation. The changes in the early-warning rankings are mainly related to the continuous coordination of regional population, economic and social development, and the environment in the past few years. In addition, in the longitudinal comparison, the ecological security warning values in 2017 and 2018 were remarkably higher than in 2011. The LD growth rate was the highest at 0.2422, indicating that the ecological environment of Qinghai’s alpine agricultural mountainous area was gradually improving and showing dynamic changes. However, in the past 8 years, PA has the best eco-environmental early-warning value of 0.5308–0.6448, and most of its ecological security levels are from III to IV. In 2018, although the overall ecological security level increased to level IV, which accounted for three districts and counties, none of the agricultural mountainous areas reached the ecological safety level of V, indicating that the overall ecological security level of the Qinghai alpine agricultural mountainous areas still has room for improvement.

### 4.3. Dynamic Changes in Ecological Security Early-Warning Values at Different Levels

#### 4.3.1. Dynamic Changes in the Population Warning Layer

**Figure 3:**
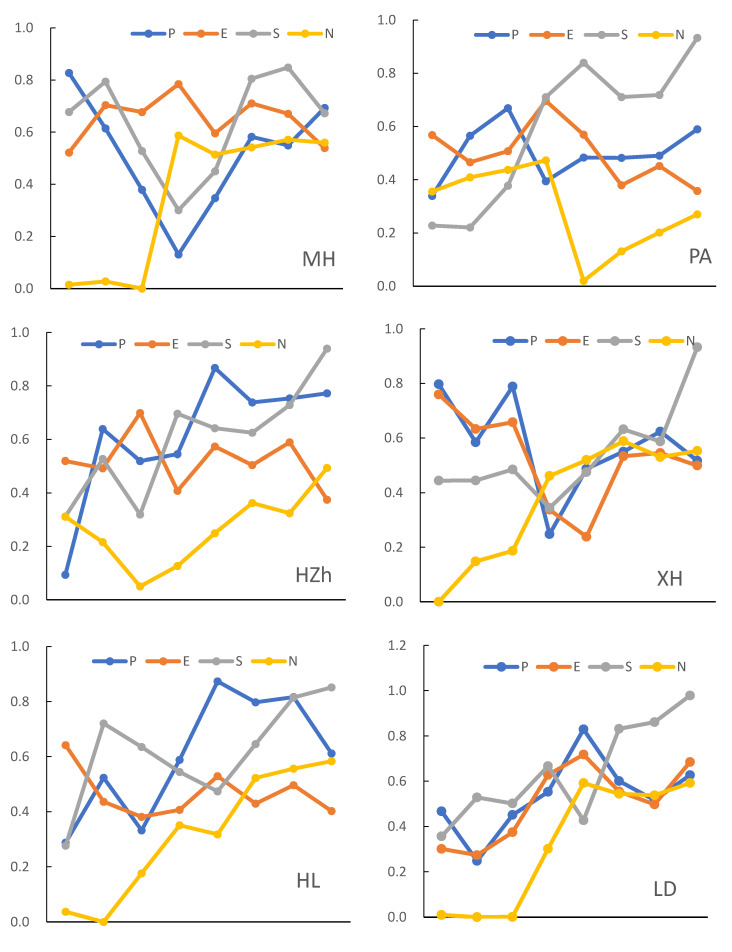

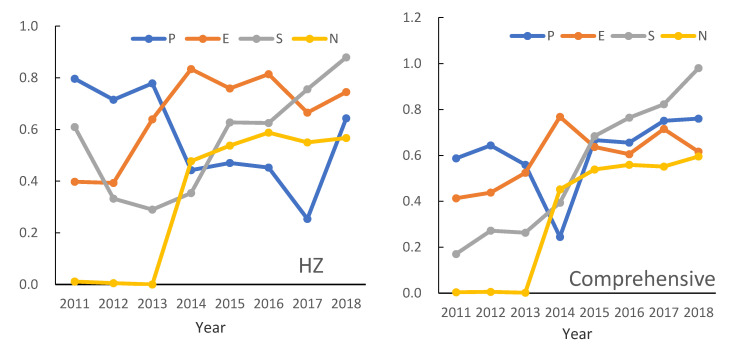
Changes in ecological security early-warning values of various districts and counties by target level and comprehensive value from 2011 to 2018. (The “comprehensive value” is calculated according to the process of 3.1 after averaging the index values of the seven districts and counties).

In summary, from 2011 to 2018, changes in the population early-warning layer in the Qinghai alpine mountainous areas were divided into three stages, and the characteristics of dynamic change show a “V” (Figure 3). In the first stage (rising stage), the early-warning value increased from 0.5870 in 2011 to 0.6470 in 2012. This increase was mainly due to the increase in the proportion of education and employment in the agricultural mountainous areas, which promoted the population early-warning layer. In the second stage (declining stage), from 2012 to 2014, there was a sharp decline in the early-warning value (0.2440), which was caused by the sudden increase in the population during this period. In the third stage (rising stage), from 2014 to 2018, the early-warning value again increased to 0.7600, indicating that population, education, employment and other aspects gradually increased, which helped the overall advancement of the population warning layer and the improvement of the ecological environment.

From different regions, the early-warning values of XH and HZ have fluctuated greatly (Figure 3). HZh, HL, LD, HZ are all four stages, in which LD and HZ early-warning values rise to varying degrees after falling in 2011–2012, while HL and HZh show a broken line fluctuation rising state, and both reach the peak in 2015. This fluctuation is due to the dramatic changes in population, education and employment in these counties over the past eight years, and the population warning level is unstable. On the warning level of population, the relative law of MH is “V” shaped. From 2011 to 2014, it showed a straight-line downward trend, and then the fluctuation rose to 0.6727 in 2018. Indicating that the population, employment and education level changed significantly in the later period. However, PA has three stages (“rise fall rise” stage). After a rapid rise in 2011–2013, PA showed a straight-line decline in 2014, and then rose slowly in 2015. Although it is not as good as the above-mentioned counties in the change stage, there are also obvious fluctuations, large amplitude and unstable overall situation. In contrast, XH has five stages, with the most frequent fluctuations. The results show that the fluctuation of the population warning layer is obvious and the state is unstable, which is mainly related to population change, employment rate, education level and other factors. To sum up, it is necessary to increase human capital investment and strengthen the ability to get rich and self-development. We should vigorously develop vocational education, organize and carry out effective skills training, improve the cultural quality of farmers in mountainous areas and promote the employment rate.4.3.2 Dynamic changes in the ecological environment early-warning layer.

In general, the change in the eco-environmental early-warning layer from 2011 to 2018 can be divided into three stages. In the first stage (the ascent stage), from 2011 to 2014, the early-warning value rose from 0.4133 to 0.7678. This indicates that the intensity of fertilizer application and natural disasters in the mountainous area of Qinghai province in the course of agricultural production have been weakened, and the pressure on the ecological environment has been alleviated to some extent. The second stage (decline stage), specifically, 2014–2016, decreased from 0.7678 to 0.6051, with a decrease rate of 21.19%. The main reason for this decline is that in recent years, the intensity of the use of chemical fertilizers and pesticides has increased remarkably, and the number of natural disasters has increased, leading to pressure on the ecological and environmental warning layer. In the third stage (the fluctuation stage), after a small recovery in 2017, the warning value dropped by 13.80% in 2017–2018. Over the past year, natural disasters and human disturbances have been the main reasons for the decline in the overall value of the ecological environment. This result shows that if it is necessary to ensure the stable and coordinated development of this layer, in addition to controlling the use of pesticides and fertilizers, it is also necessary to strictly control the impact of human production and living in the environment and to develop effective prevention and control measures for natural disasters in mountainous areas.

From the perspective of district and county, the early-warning layer of the ecological environment has fluctuated greatly. After reaching the peak in 2013, HZ presented a “broken line” trend of slow rise for 8 years and then fell and recovered rapidly. The rate of natural disasters has become a key factor affecting the fluctuations. After falling in 2012, PA rose in 2014. XH and HZh showed marked fluctuations, reaching the lowest value (0.2386) in 2015 and the highest value (0.6986) in 2013, respectively. The fluctuations of MH and HL are relatively stable. Among them, MH decreased gradually after reaching a peak in 2014. The HL showed a “U” shape and fell to its lowest point in 2013. The changes in these two stages are mainly related to the intensity of fertilizer and pesticide application, the incidence of natural disasters, human disturbance index and other factors. The ecological alert value of LD rose from 0.3009 in 2011 to 0.7179 in 2018. The results show that the eco-environmental early-warning layer of LD is more stable than that of other districts and counties, and there is strong coordination between social and economic development and eco-environmental quality.

#### 4.3.2. Dynamic Changes in the Socioeconomic Warning Layer

In general, the early-warning value of Qinghai’s alpine agricultural mountainous areas showed a linear growth trend in the past eight years (y = 0.1215X − 0.0032 R^2^ = 0.9541), reaching the highest value in 2018 (0.9798). This result shows that during this period, the social economy maintained rapid growth, the per capita net income of farmers increased year by year, and the proportion of economic density, economic output per unit of land and environmental protection investment in GDP also increased remarkably. Therefore, the overall state index shows an upward trend, and the social economy and ecological environment have developed harmoniously. From the perspective of districts and counties, HZ and LD show a folding fluctuation, both reaching the peak in 2018 while HL presents a “U” shaped state from 2011 to 2015, which increases remarkably in the later period and reaches the maximum in 2018. The socioeconomic warning values of MH and XH showed irregular “V” fluctuations, and both dropped to the lowest value in 2014. The socioeconomic warning values of PA and HZh are more stable than those of other districts and counties, showing a trend of slow decline and gradual rise. This result shows that during the 12th Five-Year Plan period, Qinghai province emphasizes green development, attaches importance to ecological function zone construction and strengthens environmental protection publicity. With the steady development of the social economy, it promotes the improvement of the ecological environment.

#### 4.3.3. Dynamic Changes in the Early-Warning Layer of Natural Resources

From 2011 to 2018, the comprehensive change trend of the early-warning layer of natural resources showed a slow rise, among which the fluctuation was large in 2013–2014 and reached the highest value in 2018 (0.5953). Due to the increased environmental protection efforts of the government, the per capita forestland area, per capita cultivated land area, per capita grain output, forest coverage rate and other indicators of this level basically increased year by year, which affected the overall trend of this level of early-warning. In terms of districts and counties, PA and HZh first decreased and then increased in 2014 and 2013, respectively. The fluctuations for the rest of the districts and counties in terms of the early-warning of natural resources were somewhat larger. The downward trend between districts and counties is mainly due to the decrease in indicators such as grain output per capita and forestland area per capita from 2014 to 2018. The results show that the natural resource layer promotes the population, life, the economy and the ecological environment.

In summary, from the dynamic analysis of the four levels, it can be concluded that the change in the ecological security early-warning value of the subtarget level in the Qinghai alpine agricultural mountainous area generally fluctuated and increased in 2011–2018. The changes in the districts and counties are quite different, and the ecological early-warning levels at all levels have shown an improvement from level I to V. The population early-warning layer and the ecological environment early-warning layer show a relatively large change, and the factors of each layer are still in the running-in stage. The social and economic early-warning layer and the natural resource early-warning layer are gradually developing well. This result shows that socioeconomic and natural resource factors play a leading role in improving the level of ecological security, which promotes the improvement of the level of ecological security, while the population layer has a smaller inhibitory effect on the overall level of ecological security. The factors of the ecological environment layer are still in a stage of harmony, which has low inhibition and promotion effects on the level of ecological security. Therefore, the ecological security early-warning level of the Qinghai alpine agricultural mountainous area has improved overall, and the level of economic and social development has generally improved.

### 4.4. Ecological Security Early-Warning Analysis Based on the GM (1, 1) 

According to the steps in the research method, first accumulate the original data $X^{\left(0\right)}$: $x^{\left(1\right)}(k)=\sum_{m=1}^{k}x^{\left(0\right)}\left(m\right)$ (*k* = 1,2, …, 8) where the original data is the result calculated according to steps 2–5. It is obtained as:

$$X^{\left(1\right)}=\left(x^{\left(1\right)}\left(1\right),x^{\left(1\right)}\left(2\right),\ldots ,x^{\left(1\right)}\left(8\right)\right)\;=\;(0.1893,\;0.4798,\;0.8303,\;1.4071,\;2.1271,\;2.9045,\;3.7092,\;4.5863);$$

Secondly, Construction of the accumulation matrix *B* and the constant vector $Y_{n}$:

$$Y=\left[\begin{array}{c} x^{\left(0\right)}(2)\\ x^{\left(0\right)}(3)\\ x^{\left(0\right)}(4)\\ x^{\left(0\right)}(5)\\ x^{\left(0\right)}(6)\\ \begin{array}{l} x^{\left(0\right)}(7)\\ x^{\left(0\right)}(8) \end{array} \end{array}\right]=\left[\begin{array}{c} 0.2905\\ 0.3505\\ 0.5768\\ 0.7200\\ 0.7774\\ \begin{array}{l} 0.8047\\ 0.8771 \end{array} \end{array}\right],B=\left[\begin{array}{cc} -z^{\left(1\right)}(2) & 1\\ -z^{\left(1\right)}(3) & 1\\ -z^{\left(1\right)}(4) & 1\\ -z^{\left(1\right)}(5) & 1\\ -z^{\left(1\right)}(6) & 1\\ \begin{array}{l} -z^{\left(1\right)}(7)\\ -z^{\left(1\right)}(8) \end{array} & \begin{array}{l} 1\\ 1 \end{array} \end{array}\right]=\left[\begin{array}{cc} -0.3346 & 1\\ -0.6551 & 1\\ -1.1187 & 1\\ -1.7671 & 1\\ -2.5158 & 1\\ \begin{array}{l} -3.3069\\ -4.1478 \end{array} & \begin{array}{l} 1\\ 1 \end{array} \end{array}\right]$$

Determination of the least-squares solution to the grey parameter vector, we obtain

$$\hat{\Phi }={\left[\hat{a},\hat{b}\right]}^{T}={\left(B^{T}B\right)}^{-1}B^{T}Y=\left[\begin{array}{c} -0.150207\\ 0.33103771 \end{array}\right]\;\hat{a}=-0.150207,\;\hat{b}=0.33103771$$

The standard form of the grey difference equation is as follows:

$$x^{\left(0\right)}(k)-0.150207z^{\left(1\right)}(k)=0.33103771$$

The time–response function of the GM (1, 1) model is as follows:

$${\hat{x}}^{\left(1\right)}\left(k+1\right)=\left[x^{\left(1\right)}\left(1\right)-\frac{\hat{u}}{\hat{a}}\right]e^{-\overset{⌢}{a}k}+\frac{\hat{u}}{\hat{a}}\;=\;2.3931768e^{0.150207k}-2.2038768$$

The recovered data $x^{\left(0\right)}(k+1)$ can be retrieved by the inverse accumulated generating operation:

$${\hat{X}}^{\left(0\right)}=\left({\hat{x}}^{\left(0\right)}(1),{\hat{x}}^{\left(0\right)}(2),\ldots ,{\hat{x}}^{\left(0\right)}(8)\right)\;=\;(0.1893,\;0.3879,\;0.4507,\;0.5238,\;0.6087,\;0.7073,\;0.8220,\;0.9552)$$

On this basis, the accuracy of the model is tested: the posterior error test results in *p* = 0.7647 (qualified), *C* = 0.3788 (good), and the overall accuracy of the model is medium; The residual test results: Relative Error Value < 20%, Stage Ratio Deviation Value (a) < 0.2. Therefore, the accuracy of the model can be used for the next prediction.

**Figure 4:**
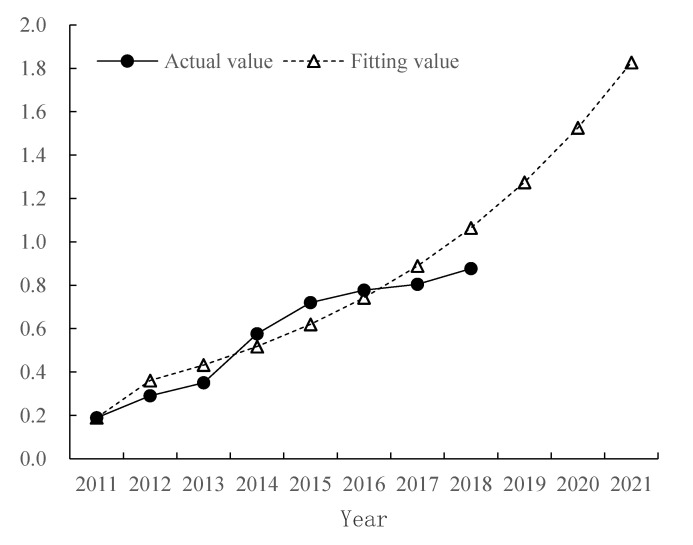
Comparison of actual value and predicted value of ecological security early-warning index in the Qinghai alpine agricultural mountainous area.

The prediction results show the overall development trend of ecological security in Qinghai Alpine agricultural mountainous areas from 2019 to 2021 (Figure 4). The prediction formula is ${\hat{x}}^{\left(0\right)}(k+1)=-a\left(X^{\left(0\right)}\left(1\right)-\frac{b}{a}\right)e^{-ak}=0.359472e^{0.150207k}$, and the future forecasted value will show a linear increasing trend (y = 0.1945x + 0.9107, R^2^ = 0.9981). According to Figure 4, the early-warning value of ecological security in the Qinghai alpine agricultural mountainous area showed an increasing trend each year. The early-warning degree will continue to change to the “ideal” state from 2019 to 2021, and the ecological environment status will continue to improve compared with the “good” state in 2016.

According to Figure 4, the ecological security early-warning value of the Qinghai alpine agricultural mountainous area shows an increasing trend each year. The early-warning degree will continue to change to the “excellent” state from 2019 to 2021, and the ecological environment status will continue to improve compared with the “good” state in 2016. The main reason for this change is that social and economic development has continued, and per capita GDP, farmers’ net incomes, employment rate, and education level as a whole have improved from 2011 to 2015, all of which have become important factors that promote the development of the local ecological security level toward “excellent”. However, in the province’s twelfth and thirteenth five-year environmental protection plans, we will strengthen ecological environmental protection and construction, and build a green and low-carbon recycling industry system. The emissions of “three wastes” will continue to decrease, and the overall ecological safety level will be improved year by year. Especially after 2020, with the comprehensive completion of a well-off society, the coordination between the subsystems of population, socioeconomics, ecological environment, and natural resources will have reached the best level. This result means that, at this time, the ecosystem functions are complete, the structure is complete, and the ability of the alpine mountainous area to resist external disturbances and recovery is enhanced. The level of local socioeconomic development has improved, and various policies are conducive to ecological environmental protection, achieving the ideal state of coordinated development of the social economy and natural ecological environment, which is suitable for people to live and work in peace. Although the ecological environment is gradually developing well, against the background of a fragile and arid natural environment, sustainable development can last for a long time only with the comprehensive support of various policies.

### 4.5. Ecological Security Early-Warning Spatial Differentiation Characteristics

**Figure 5:**
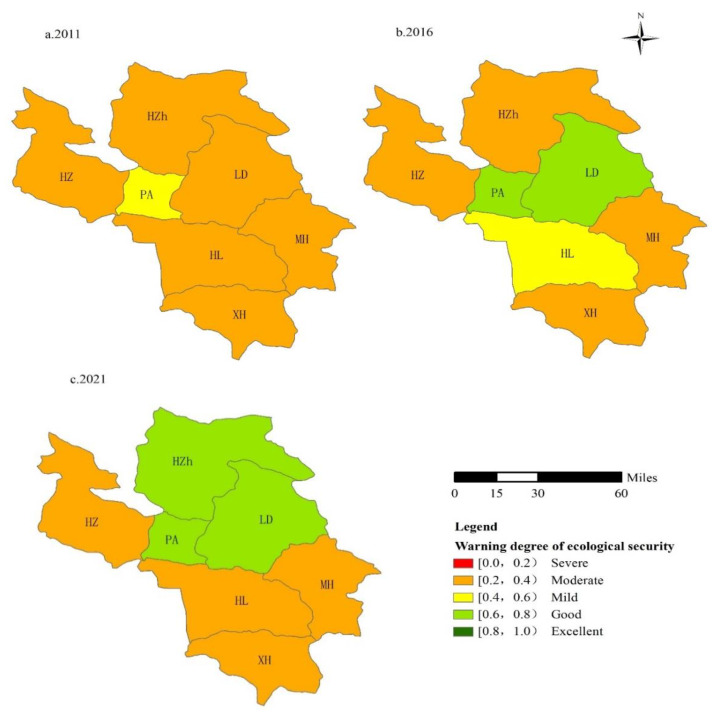
Regional distribution of ecological security warnings in the Qinghai alpine agricultural mountainous area in 2011, 2016 and 2021 (The letters “a”, “b”, and “c” represent the spatial differentiation pattern of the three research time periods of the initial, intermediate, and forecast (final) respectively).

On the whole, in 2011, the ecological security of various districts and counties in Qinghai’s alpine agricultural mountainous area basically showed a low level; that is, the early-warning risk was high, and the ecological quality was poor. In 2016, the ecological alert level showed a high trend in the northeast and southeast, and a low trend in the central region. It is expected that in 2021, the early-warning level of ecological security in the Northeast will increase remarkably, and the early-warning level of ecological security in the Southeast will be better than the previous two periods. The characteristics of spatial differentiation also present a pattern of high in the north and low in the south. Among them, PA and LD in the northeast have the best ecological environment. In 2011, the overall ecological security was basically at level II, and some districts and counties were at level III. In 2016, the ecological safety of all districts and counties generally reached level III, and individual districts and counties remained at level II. In 2021, the proportion of districts and counties with ecological security status at level IV will increase, and the proportion of districts and counties at levels II and III will decrease remarkably. The results show that while the overall ecological environment of Qinghai’s alpine agricultural mountainous areas has improved, internal differences have also increased. The socioeconomic development status and ecological environment improvement speeds of counties and counties in the northeast are higher than those in the western region (Figure 5).

Specifically, in 2011, the eco-safety early-warning status of all districts and counties in Qinghai’s alpine agricultural mountainous area was level II or III. That is, the level of ecological security was low, the ecological pressure was high, the level of socioeconomic development was relatively backward, and the early-warning risk was high. Except for PA in a mild warning state, other districts and counties were in a moderate warning state, including LD, MH, HZh, XH, HL, and HZ. The main reason for this condition is that the population growth rate of all districts and counties in 2011 was rapid, the per capita woodland area and per capita arable land area were generally low, farmers’ awareness of ecological and environmental protection was weak, and the application intensity of chemical fertilizers and pesticides was relatively high, which led to a sharp decline in environmental quality in the mountainous areas. In addition, natural conditions are fragile and dry, natural disasters occur frequently, backward economic and social development cannot drive the construction of the ecological environment, and the overall ecological security warning level is low.

In 2016, the ecological safety early-warning index of all districts and counties increased rapidly. Among them, the MH, HZ, XH, and HZh ecological safety early-warning index rose from a moderate early-warning to a light early-warning, and the LD ecological security early-warning status rose from a moderate early-warning to a good one. This result is mainly based on the strategic position of Qinghai Province in the construction of national ecological civilization. During the “Thirteenth Five-Year Plan” period, we will comprehensively carry out rural environmental improvement, strengthen environmental monitoring and environmental law enforcement, and effectively promote the coordinated development of economic society and environmental protection. Therefore, the level of early-warning of ecological security has been improved.

According to the forecast, in 2021, the prewarning level of ecological security in various districts and counties will increase remarkably; however, the internal level differences will gradually become prominent. The ecological security level will range from level II to level V. The northeast region will be in the leading position with HZh, LD, and PA. Mainly due to the rapid economic and social development during this period, the comprehensive construction of a well-off society will promote the improvement of the ecological environment. This will reflect the implementation of various ecological protection policies by the government, the promotion of green agricultural products, the increase in green areas, and the convenience of transportation. The improvement of infrastructure and the enhancement of people’s concept of ecological protection will provide guarantees for local ecological safety. In addition, Xining, the capital of Qinghai Province, was successfully named the “Fifth National Civilized City”, which has a direct driving effect on surrounding districts and counties. Therefore, it can be predicted that no major ecological crisis will occur in these districts and counties in the future. The ecosystem functions are sound, the level of social and economic development is high and good, and the coordinated development of population, social economy, ecological environment and natural resources is realized. This area can be used as a standard for other counties to promote the construction of ecological civilization. HZ and XH counties have low levels of ecological safety early-warning, from mild early-warning to moderate early-warning, with moderate early-warning being the main one. However, the economic and social development of these counties is not the same. Xining is part of the provincial capital of Xining. The main reason is that population growth is too fast, resulting in a decrease in per capita natural resources. XH lacks coordinated development in the social economy and ecological environment early-warning level. This result shows that protecting the ecological environment does not mean that the economy cannot be developed further; however, it cannot be based on excessive dependence on natural resource consumption and environmental pollution.

Therefore, the overall level is in the middle, and the scores of ecological security early-warning are low, which leads to its ecological security ranking in the backward position among the seven districts and counties.

## 5. Discussion

### 5.1. Reliability and Limitations of the Early-Warning Model

The study area is among the areas with fragile ecological environments in China, and it is also among the areas with heavy tasks, great difficulty and deep degrees of poverty in China’s poverty alleviation and development. The vast majority of poor farmers live in high-altitude areas with arid and water shortages in the east, relatively backward economic and social development, with the ability to grow cold-resistant crops. At the same time, the region plays an important role in the overall promotion of Qinghai’s “ecological province” development strategy. Therefore, the future ecological security of the region will partly determine whether this development goal can be achieved. Although the results of the grey warning model show reliable accuracy and can accurately reflect the actual situation of the study area, there are still some defects in this study. First, the current research on ESE is relatively weak, and there is no quantitative standard to determine the early-warning level, especially for the Qinghai Tibet Plateau, which affects the comparability of different research results. Second, the grey early-warning model shows only that it has a high ability for short-term or medium-term prediction; however, it cannot accurately predict long-term early-warning levels. Although scenario analysis is widely used in change prediction, and future trends can be fully discussed [101,102], we choose a trend extrapolation method, i.e., the grey model, because there are many important scenarios for which parameters cannot be obtained in ecological security assessment. In contrast, the grey warning model established by us has reliable and feasible accuracy, which can meet the demand of future ecological security change prediction [103]. Finally, ecological security is complex and dynamic, and some index data are difficult to obtain. Therefore, the indicator system must be improved. For example, we have not considered the impact of pollution and policy on the results of early-warning. Therefore, in future research, we should gradually improve the evaluation system.

### 5.2. Analysis of Ecological Security Status and Influencing Factors

The results show that the ecological security situation will be gradually improved in the next few years; however, the development level of ecological security is unbalanced in terms of space. From the evaluation results, in 2011 and 2016, the areas with poor ecological security are mainly concentrated in the southwest. The ecological environment of the Qinghai Tibet Plateau is mainly affected by climate change, land desertification, human activities and natural environment changes [104]. In addition, the future (2021) ecological security situation will be improved remarkably in Northeast China, and there will still be ecological risk problems in Southwest China; that is, the ecological risk areas will still be mainly distributed around XH in the South and HZ in the West. The ecosystems in these areas are threatened because the level of economic development in these areas is lower than that in other areas, and the prominent manifestations of these areas are severe land degradation, aggravation of soil and water loss, and the increasing threat of biodiversity [105,106]. In addition, the ecological security situation in the western region is poor, and it has been a “middle police” state for many years, which indicates that the environmental pressure and habitat fragmentation caused by population aggregation and frequent natural disasters are also important factors affecting ecological security. Although the overall ecological security situation will be improved, it is still necessary to take effective measures and formulate reasonable planning to actively improve the ecological security situation in the western region. In addition, there are some differences in the results of ecological early-warning. The main reason is that although the early-warning results at the county level can reflect the overall situation of the county, the basis is the maximum value, minimum value and average value of each evaluation index. In other words, the ecological security status at the county level often depends on the worst situation of regional indicators, reflecting the overall situation of the region. However, to obtain the best research results for the agricultural mountainous areas, the author believes that the use of smaller-scale data, such as on the rural level, should be more representative and accurate.

## 6. Conclusions 

### 6.1. Conclusions

In this paper, an ecological security evaluation method is applied to analyze the ecological security of the Qinghai alpine agricultural mountainous areas and to study spatial patterns. The method and model used in this paper can also be applied to other alpine agricultural mountainous areas to evaluate the impact of human activities on the ecological environment of alpine agricultural mountainous areas.

The results show that the ecological environment of the Qinghai alpine agricultural mountainous area has been gradually improved, and the ecological security early-warning value of each district and county in 2018 is greater than that in 2011. Although the ecological environment has improved in the past five years, from the perspective of the ecological security early-warning state in 2015, the ecological security early-warning level of most areas is still at the second and third levels, which indicates that the ecological environment is in a general state, the ecosystem structure has slightly changed, and the service function has been degraded to a certain extent; however, the basic function can still be maintained. From the spatial pattern perspective, the ecological early-warning degree of the Qinghai alpine agricultural mountainous area presents high characteristics in the north and low characteristics in the south, and the difference in the north is reduced. The model simulation results show that the early-warning value of ecological security in the Qinghai Tibet Alpine agricultural mountainous area will slowly increase in the next few years, which is similar to the prediction result obtained by Chen Yun et al. [107]. It is concluded that the situation of ecological security is still grim in the future, and the management and regulation of ecological security should be strengthened in the future. In addition, in a similar case, the researchers focused on the risk assessment and early-warning mechanism related to the development of ecotourism in Tianmu Mountain, China, and found early warning signs of ecosystem degradation in Jiuzhaigou Nature Reserve in China [108,109].

Based on these findings, this paper calls for an interdisciplinary research agenda at the intersection of land-use change and ecological risk modeling in agricultural mountainous areas. The problems of socioeconomic and ecological protection in agricultural mountainous areas, which are lacking in the process of local planning, should be solved through discussion in specific local departments. In addition, increasing land-use change data provide large-scale and macro-level insights for the ecological economy.

Further research can explore the impact assessment and ecological risk modeling of land-use change in specific areas (Qinghai Tibet Alpine agricultural mountainous areas) to better support local policies. It is worth noting that although this study provides evidence that is consistent with that of previous studies, it is still unable to draw a definite conclusion that the ecological environment of the whole Qinghai Tibet Plateau tends to have improved. In contrast, the ecological security of alpine agricultural mountainous areas depends on the joint action of multiple and complex factors rather than on a single population change or labor flow.

### 6.2. Recommendations

The ecological environment of the Qinghai alpine agricultural mountainous areas has generally improved; however, it is still far from reaching the “excellent” state. There are marked gaps in ecological security levels within the regional interior. To better optimize and enhance the ecological environment in the mountainous areas, separate measures can be taken for areas in different security states based on actual conditions.

For northeastern regions with higher ecological security levels, such as PA, it is necessary to further improve ecosystem service functions, pay attention to the governance and protection of the ecological environment, and strengthen the development of recycling technologies.

At the same time, we will adopt reasonable population policies, optimize the industrial structure, develop a green circular economy, coordinate the development and utilization of land resources, explore new ideas for the development of ecological agriculture, and reduce the use of pesticides and fertilizers. For western districts and counties (HZ and XH) with ecological security levels II and III, the relationship between economic development and environmental protection should first be coordinated according to the actual ecological carrying capacity of the region.

Ecological restoration or reconstruction should be carried out in some degraded areas. Second, we should strictly implement environmental protection standards and a total pollutant emission control system and improve ecological environment monitoring as well as early-warning and supervision and law enforcement systems. The protection and management of the Huangshui River basin should be strengthened, the return of farmland to forests should be implemented, the coverage of forest trees should be increased, and soil erosion should be prevented. Finally, we should optimize the industrial structure, develop a circular economy, and develop green agriculture to increase the utilization of natural resources. We should focus on interregional coordination, quickly establish and improve the ecological early-warning system in the alpine mountainous areas, and improve the ability to respond to ecological emergencies. We will increase publicity, advocate ecological ideas, and raise awareness of the need for environmental protection.

Ecological security early-warning in alpine agricultural mountainous areas is a complex systematic project comprising many factors. The conclusion of this work is directly related to the protection of the alpine region and even the global ecological environment, and the need for a reasonable index system has an important impact on the results. Because of data limitations, this research can be deepened and improved further. In particular, the study of alpine areas is not mature and needs further improvement. In the future, a larger scale and a longer time span can be selected, which will better reveal the changes in ecological security and provide effective evidence for future ecological security regulation.

## Figures and Tables

**Table 1:** The ecological security early-warning criterion.

Comprehensive Early-Warning Value	Ecological Safety Level	Ecological Security Early-Warning Status
[0.0, 0.2)	I	Huge alarm (Huge)
[0.2, 0.4)	II	Moderate warning (Moderate)
[0.4, 0.6)	III	Light warning (Light)
[0.6, 0.8)	IV	Good
[0.8, 1.0)	V	Excellent

**Table 2:** Accuracy levels of the grey model (GM) (1, 1) [99,100].

Accuracy Level	*p*	*C*
Excellent	*p* ≥ 0.95	*C* ≤ 0.35
Good	0.80 ≤ *p* < 0.95	0.35 < *C* ≤ 0.50
Accepted	0.70 ≤ *p* < 0.80	0.50 < *C* ≤ 0.65
Failed	*p* < 0.70	0.65 > *C*

**Table 3:** Model accuracy test results.

District	Posterior Error Ratio		Stage Ratio Deviation Value		Accuracy
	*C*	*p*	Relative Error Value (%)	Stage Ratio Deviation Value	
**MH**	0.3923	0.7647	3.8	0.048	Accepted
PA	0.5249	0.7059	2.6	0.029	Accepted
HZ	0.4045	0.7059	2.4	0.032	Accepted
XH	0.4393	0.7059	1.6	0.020	Accepted
HL	0.46	0.7059	4.7	0.056	Accepted
LD	0.4535	0.7059	5.5	0.061	Accepted
HZh	0.4443	0.7647	5.8	0.058	Accepted

**Table 4:** The system of indexes used for the Qinghai alpine agricultural mountainous area ecological security early-warning.

Target Layer	Indicator Layer	Calculation Method	Direction	Type Weight	Comprehensive Indicator Weight
Population early warning system (P)	Population density (person/km^2^)	Population/area	−	0.1807	0.0323
	Natural growth rate of population (‰)	(Annual births-annual deaths)/Annual average∙1000‰	−	0.2297	0.0411
	Nine-year compulsory education consolidation rate (%)	Number of graduates/enrollment (including normal migrant students) 100%	+	0.3843	0.0687
	Proportion of rural employees (%)	Total rural employment/labor resources	+	0.2053	0.0367
Ecological environment early-warning system (E)	Chemical fertilizer application intensity (kg/hm^2^)	Fertilizer application amount/arable land area	−	0.2103	0.0580
	Pesticide application intensity (kg/hm^2^)	Amount of pesticide application/arable land area	−	0.1243	0.0343
	Natural disaster incidence rate (%)	(Disaster area/Affected area)∙100%	−	0.2030	0.0560
	Human interference index (%)	(Cultivated land area + Construction land area)/Regional land area 100%	−	0.1921	0.0530
	Domestic sewage treatment rate (%)	Domestic sewage treatment capacity/Total domestic sewage discharge·100%	+	0.0796	0.0220
	Domestic waste treatment rate (%)	Total waste processed/total waste generated	+	0.1907	0.0526
Socioeconomic early warning system (S)	Per capita GDP (10,000 yuan/person)	GDP/Total population at the end of the year	+	0.1825	0.0470
	Per capita net income of farmers (yuan)	(Total income of rural households—Expenditure on household operating expenses—Depreciation of productive fixed assets—Taxes and surrendering contract fees−Survey subsidies)/Permanent population of rural households	+	0.1847	0.0476
	Proportion of tertiary industry in GDP (%)	Tertiary industry output value/GDP∙100%	+	0.1420	0.0366
	Economic density (10,000 yuan/km^2^)	GDP/Area	+	0.2027	0.0522
	Proportion of environmental protection investment to GDP (%)	Investment in environmental protection/GDP·100%	+	0.2018	0.0520
	Economic output per unit of land (10,000 yuan/km^2^)	Total output value of agriculture, forestry, animal husbandry and fishery/Regional land area	+	0.0863	0.0222
Natural resources early-warning system (N)	Woodland area per capita (hm^2^/person)	Woodland area/Total population at the end of the year	+	0.3112	0.0896
	Per capita arable land area (hm^2^/person)	Arable land/Total population at the end of the year	+	0.2842	0.0818
	Per capita output of grain (kg/person)	Total grain production/Total population at the end of the year	+	0.2422	0.0697
	Percentage of forest cover (%)	Forest area/Area·100%	+	0.1625	0.0468

**Table 5:** Ecological security early-warning values of districts and counties from 2011 to 2018.

Year	Item	District						
		MH	PA	HZ	XH	HL	LD	HZh
2011	EWV	0.3460	0.5441	0.3377	0.3992	0.3187	0.3868	0.3913
	ESL	II	III	II	II	II	II	II
2012	EWV	0.3311	0.5308	0.3957	0.3737	0.2966	0.4344	0.4157
	ESL	II	III	II	II	II	III	III
2013	EWV	0.3361	0.5549	0.4218	0.3680	0.2918	0.4461	0.4345
	ESL	II	III	III	II	II	III	III
2014	EWV	0.3917	0.6220	0.4037	0.3836	0.3371	0.5343	0.5320
	ESL	II	IV	III	II	II	III	III
2015	EWV	0.3952	0.5675	0.4584	0.3991	0.3959	0.6179	0.5761
	ESL	II	III	III	II	II	IV	III
2016	EWV	0.4220	0.5996	0.4495	0.4002	0.3905	0.6050	0.5845
	ESL	III	III	III	III	II	IV	III
2017	EWV	0.4491	0.6354	0.4766	0.3937	0.4075	0.6116	0.5838
	ESL	III	IV	III	II	III	IV	III
2018	EWV	0.4273	0.6448	0.4761	0.4237	0.3967	0.6290	0.6008
	ESL	III	IV	III	III	II	IV	IV

Note: The ecological security early-warning value is abbreviated as “EWV”, the security level is abbreviated as “ESL”.
